# Phenotype testing, genome analysis, and metabolic interactions of three lactic acid bacteria strains existing as a consortium in a naturally fermented milk

**DOI:** 10.3389/fmicb.2022.1000683

**Published:** 2022-09-23

**Authors:** Javier Rodríguez, Lucía Vázquez, Ana Belén Flórez, Baltasar Mayo

**Affiliations:** ^1^Departamento de Microbiología y Bioquímica, Instituto de Productos Lácteos de Asturias (IPLA), Consejo Superior de Investigaciones Científicas (CSIC), Villaviciosa, Spain; ^2^Instituto de Investigación Sanitaria del Principado de Asturias (ISPA), Oviedo, Spain

**Keywords:** lactic acid bacteria, *Lactococcus lactis*, *Lactococcus cremoris*, *Lactiplantibacillus plantarum*, starters, naturally fermented milk, consortium, genomics

## Abstract

This work reports the characterization of three lactic acid bacteria (LAB) strains –*Lactococcus lactis* LA1*, Lactococcus cremoris* LA10, and *Lactiplantibacillus plantarum* LA30– existing as a stable consortium in a backslopping-inoculated, naturally fermented milk (NFM). This study aimed at uncovering the biochemical and genetic basis of the stability of the consortium and the cooperativity among the strains during milk fermentation. All three strains were subjected to phenotyping, covering the utilization of carbohydrates, enzyme activity, and antibiotic resistance. The strains were grown in milk individually, as well as in all possible combinations, and the resulting fermented product was analyzed for sugars, organic acids, and volatile compounds. Finally, the genomes of the three strains were sequenced and analyzed for genes associated with technological and safety properties. As expected, wide phenotypic diversity was seen between the strains. *Lactococcus cremoris* LA10 was the only strain to reach high cell densities and coagulate milk alone after incubation at 22°C for 24 h; congruently, it possessed a gene coding for a PrtP type II caseinolytic protease. Compared to any other fermentation, acetaldehyde concentrations were greater by a factor of six when all three strains grew together in milk, suggesting that its production might be the result of an interaction between them. *Lactococcus lactis* LA1, which carried a plasmid-encoded *citQRP* operon, was able to utilize milk citrate producing diacetyl and acetoin. No genes encoding virulence traits or pathogenicity factors were identified in any of the strains, and none produced biogenic amines from amino acid precursors, suggesting them to be safe. *Lactiplantibacillus plantarum* LA30 was susceptible to tetracycline, although it harbors a disrupted antibiotic resistance gene belonging to the *tetM/tetW/tetO/tetS* family. All three strains contained large numbers of pseudogenes, suggesting that they are well adapted (“domesticated”) to the milk environment. The consortium as a whole or its individual strains might have a use as a starter or as starter components for dairy fermentations. The study of simple consortia, such as that existing in this NFM, can help reveal how microorganisms interact with one another, and what influence they may have on the sensorial properties of fermented products.

## Introduction

Fermentation is perhaps the oldest milk preservation technique. Lactic acid bacteria (LAB) lie behind the spontaneous fermentation of milk; these promote its acidification, inhibiting the growth of spoilage and pathogenic microorganisms, and causing coagulation when the pH approaches the isoelectric point of casein (≈pH 4.6; Sharma et al., 2021). The enzymes of LAB also partially digest lactose and milk proteins, contributing to the bioavailability of sugars and amino acids and the formation of taste and aroma compounds (Bintsis, 2018). Two subclasses of naturally fermented milks (NFMs) can be distinguished: non-inoculated and inoculated (Robinson and Tamime, 2006). The former NFMs are made by leaving milk at room temperature until acidification causes the coagulum to appear, while the latter are manufactured by adding a portion of a previous batch to a new one –a process known as backslopping (Garofalo et al., 2020). In every transfer each LAB strain must compete with all other bacteria present and is therefore under pressure to grow quickly.

In nature, microorganisms do not live alone but in complex communities where positive and negative interactions occur (Weiland-Bräuer, 2021). Microbial interplay is mediated *via* a variety of molecular and physiological mechanisms, among which the exchange of metabolites (cross-feeding) is among the most typical (Pierce and Dutton, 2022). Trophic chains in foods enable multiple groups of organisms to survive on limited resources and under stressful conditions (Shanafelt and Loreau, 2018), such as those that reign in the acidic environments of fermented dairy foods (Oshiro et al., 2019; Sun and D’Amico, 2021). Some microbes, however, produce substances that inhibit or kill other microorganisms, impeding the development of their competitors (Barua et al., 2021). Although a plethora of microbial interactions exists, four main types are generally contemplated: competition, amensalism, commensalism and mutualism (Mayo et al., 2021; Pierce and Dutton, 2022). These interactions are not mutually exclusive, and over the manufacture and ripening of dairy products, many may occur at the same time among the different components of the microbial community (Smid and Lacroix, 2013; Mayo et al., 2021). Indeed, some communities have properties that could not be predicted from examining those of their individual members (Minty et al., 2013). They also contain genetic and functional redundancy, which contributes to their robustness (Shapiro and Polz, 2014).

Some years ago, our laboratory reported on an inoculated NFM of unknown (although Eastern) origin, to contain a bacterial consortium of just three strains (Alegría et al., 2010), one of *Lactococcus lactis* subsp. *lactis*, one of *Lactococcus cremoris* subsp. *cremoris* (formerly *Lactococcus lactis* subsp. *cremoris*), and one of *Lactiplantibacillus plantarum* (formerly *Lactobacillus plantarum*). All three strains were found present in fixed numbers (10^8^–10^9^ cfu ml^−1^ lactococci; 10^6^–10^7^ cfu ml^−1^ *L. plantarum*) in batches sampled some 18 months apart, indicating the partnership to be very stable. The main goals of the current work were to reveal the biochemical and genetic basis of the consortium stability and the interplay of the strains during milk fermentation. This knowledge could help in the rational use of the consortium as a starter in dairy and may provide fundamental knowledge for the design of more complex, multi-strain cultures. In summary, the study reports on the phenotypic properties of the individual strains, the sequencing and analysis of their genomes, and some strain–strain interactions seen to occur during their growth in milk.

## Materials and methods

### Strains and culture conditions

*Lactococcus lactis* subsp. *lactis* LA1 (*L. lactis*), *Lactococcus cremoris* subsp. *cremoris* (*L. cremoris*) LA10, and *Lactiplantibacillus plantarum* (*L. plantarum*) LA30 had been isolated from an NFM in a previous work (Alegría et al., 2010); they were the only bacteria present in that fermented milk. Unless otherwise stated, strains were cultured in M17 broth (Oxoid, Basingstoke, United Kingdom) supplemented with 0.5% glucose (GM17; lactococci) or MRS (Oxoid; *L. plantarum*) in aerobiosis at 32°C (the routine testing temperature for mesophilic LAB) for 24–48 h. When required for plates, media were solidified by adding 2% agar to the liquid version; culturing was performed under the same conditions.

### Strain identification by 16S rRNA gene sequencing

The previous identification of the strains was confirmed by PCR amplification of a major part of the 16S rRNA gene, using the universal primers 27F (5′-AGAGTTTGATCCTGGCTCAG-3′) and 1492R (5′-GGTTACCTTGTTACGACTT-3′), sequencing the amplicons, and comparing them with those in databases, as reported elsewhere (Rodríguez et al., 2022).

### Phenotyping of strains

The phenotypic profiles of the individual strains were determined *via* a battery of biochemical tests, as described below.

#### Fermentation of carbohydrates

The carbohydrate fermentation profile was assessed using the API50 CHL system (bioMérieux, Montalieu-Vercieu, France) following the supplier’s recommendations. Briefly, a single colony of each strain grown on either GM17 or MRS agar plates was suspended in 2 ml of sterile saline (0.9% NaCl) to reach a density corresponding to McFarland standard 2 (spectrophotometric equivalent of ≈6 × 10^8^ cfu ml^−1^). This suspension was then used to inoculate the CHL medium at 1% (v/v). A 180 ml aliquot of the inoculated medium were dispensed into the API50 strip wells; these were then covered with oil and the strips incubated at 32°C for 48 h.

#### Enzyme activities

Enzyme activities were measured using the commercial, semi-quantitative API-ZYM system (bioMérieux) according to the manufacturer’s instructions. Sixty-five μl of cell suspensions of isolated colonies from agar plates, corresponding to McFarland standard 5 (≈1 × 10^9^ cfu ml^−1^), were inoculated into each well of an API-ZYM strip. This was incubated for 4 h at 32°C and developed as recommended. Following the bioMérieux scale, the activity of each enzyme was expressed as 0 to ≥40 nmol of substrate hydrolyzed.

#### Antimicrobial resistance–susceptibility

The minimum inhibitory concentration (MIC) of 16 antibiotics was determined for each strain by broth microdilution using VetMIC™ plates for LAB (National Veterinary Institute of Sweden, Uppsala, Sweden). The wells were inoculated with 150 μl of a cell suspension corresponding to McFarland standard 1 diluted 1:1,000 in liquid IsoSensitest (Oxoid) for lactococci, or LSM (Klare et al., 2005) for lactobacilli (≈3 × 10^6^ cfu ml^−1^). The resistance-susceptibility of the strains was defined following the European Food Safety Authority’s (EFSA) microbiological cut-offs for *L. lactis* and *L. plantarum*/*L. pentosus* (EFSA FEEDAP Panel (EFSA Panel on Additives and Products or Substances used in Animal Feed), 2018).

#### Production of GABA and biogenic amines

The three strains were tested for the production (in culture supernatants) of γ-aminobutyric acid (GABA) from monosodium glutamate, and the biogenic amines histamine, tyramine and putrescine from tyrosine, histidine, and arginine/lysine, respectively. Strains were grown in either GM17 (lactococci) or MRS (*L. plantarum*) supplemented with 2 mM of one of the precursors. After incubation, amino acids and derivatives in supernatants were derivatised with diethyl ethoxymethylenemalonate (DEEMM) and identified and quantified by ultra-HPLCI, according to a standardized protocol (Redruello et al., 2013). *Enterococcus faecalis* V583 was used as a positive control for tyramine production.

### Growth and metabolites production in milk

UHT-treated, semi-skimmed milk (CAPSA, Siero, Spain) was inoculated with each strain singly, two by two, and all three strains together, giving rise to seven different fermentations: LA1, LA10, LA30, LA1 + LA10, LA1 + LA30, LA10 + LA30, and LA1 + LA10 + LA30. An inoculum size of ≈3 × 10^5^ cfu ml^−1^ was always used, and the incubations proceed at 22°C (which mirrors the room fermentation temperature of the NFM) for 48 h. The growth of the strains, the pH reached, and the production of organic acids and volatile compounds, were determined as described below. Unless otherwise stated, analyses were performed in triplicate.

#### Growth of the strains in milk

Bacterial counts were recorded by dissolving the inoculated milk samples in a warm 2% citrate solution and making 10-fold dilutions in saline. These dilutions were then plated onto GM17 for counting lactococci, and MRS for counting *L. plantarum*. The pH of the milk was measured using a pH-meter (Crison, Barcelona, Spain).

#### Production of organic acids

The organic acids and sugars produced or consumed during growth in milk (by both individual strains and their mixtures) were determined by Ultra High Performance Liquid Chromatography (UHPLC) following the method of Alegría et al. (2016). Briefly, compounds were separated in an ICSep ICE-ION-300 ion-exchange column (Waters, Waltham, MASS, United States), with 8.5 mN H_2_SO_4_ as the mobile phase (operating temperature 65°C, flow rate of 0.4 ml min^−1^). Sugars were identified using a Waters model 410 differential refractometer at 280 nm, and organic acids using a Waters model 996 photodiode array detector at 210 nm. The concentration of individual metabolites was obtained using calibration curves prepared with commercial standards.

#### Production of volatile compounds

Volatile compounds in the fermented milks were quantified by headspace/gas chromatography/mass spectrometry (HS/GC/MS) using an Agilent apparatus with G 1888 HS, 6890 GC and 5975B inert MSD components (Agilent Technologies, Wilmington, DE, United States), equipped with an HP-Innowax column (length 60 m, internal diameter 0.25 mm, 0 film.25 μm; Agilent). Sample preparation and gas chromatographic analysis were performed as described by Fernández et al. (2011). After incubation, 100 μl of internal standard (cyclohexanone, 0.36 mg ml^−1^) were added and these mixtures stored at −80°C until analysis. Peaks were quantified as the relative total ionic count with respect to the internal standard.

### Genome sequencing and analysis

For genome sequencing, total DNA from the three trains was extracted using the QIAmp DNA Mini Kit (Qiagen, Hilden, Germany). Sequencing libraries were prepared using the TruSeq DNA PCR-free Sample Preparation Kit (Illumina, San Diego, CA, United States), and paired-end sequenced using a HiSeq 1500 System. Reads were checked for quality with FastQC,^1^ and trimmed for quality optimization with TrimGalore.^2^ Contigs were assembled using Velvet software v.1.2.10.^3^ Genomes were annotated and analyzed using PATRIC services.^4^ Antibiotic resistance and virulence genes were further investigated by genome comparison against sequences in the Resfinder,^5^ CARD,^6^ VFDB (Virulence Factor Database; http://www.mgc.ac.cn/VFs/), and Victors^7^ databases. Whole-genome sequence data were used to ascertain the phylogenetic relationships between the sequenced strains and the type strains of *Lactococcus* and lactobacilli species by means of digital DNA–DNA hybridization (dDDH) and orthologous average nucleotide identity (orthoANI) analysis, as reported by Meier-Kolthoff and Göker (2019) and Yoon et al. (2017), respectively.

The genome sequences of all three examined strains were deposited in the GenBank database under Bioproject PRJNA876833 and BioSample accession numbers SAMN30673470 (*L. lactis* LA1), SAMN30673504 (*L. cremoris* LA10), and SAMN30673505 (*L. plantarum* LA30).

## Results and discussion

This work reports on the phenotypic and genomic characterization of three strains of different species that together form a highly stable bacterial consortium capable of producing an appealing NFM, widely-spread and consumed in households across Europe. Complex, undefined microbial communities are widely used as starters in food biotechnology, including the manufacture of cheese, and other fermented food commodities based on meat, vegetables, cereals and fish (Oshiro et al., 2019; Sun and D’Amico, 2021). Identifying their components and characterizing at phenotypic and genetic levels their stability and cooperativity properties during milk fermentation could help the rational design of multi-strain starters from a pool of genome-sequenced LAB of different origins (Minty et al., 2013; Shapiro and Polz, 2014).

### Biochemical phenotyping

After confirming the previous identification of the strains, they were subjected to a battery of phenotypic tests, including, among others, carbohydrate utilization, enzyme profiling, and antibiotic resistance. Table 1 shows the carbohydrate fermentation profiles of the three LAB strains. Wide variation was noted: among the 49 carbohydrates tested by the API-50 strips, *L. lactis* LA1 fermented 13, *L. cremoris* LA10 used only 6, and *L. plantarum* LA30 fermented 18. The enzyme activities of the strains, as determined using the API-ZYM system are summarized in Table 2. The two *Lactococcus* strains showed a reduced and weak profile, but strong acid and alkaline phosphatase activity. In contrast, *L. plantarum* LA30 showed vigorous leucine arylamidase (aminopeptidase), valine arylamidase (aminopeptidase), and β-galactosidase activity, and moderate β-glucosidase and N-acetyl-β-glucosidase activity. In agreement with the present results, wide phenotypic variations have been repeatedly reported across LAB species and strains (Siezen et al., 2010; Bayjanov et al., 2013; Moraes et al., 2013). A reduced carbohydrate fermentation profile has been reported for dairy lactococci compared to those of plant origin (Kelly et al., 2010; Laroute et al., 2017; Wels et al., 2019), but the small number of carbohydrates (only six) utilized by *L. cremoris* LA10 was surprising, suggesting it to be a “domesticated” dairy strain (Cavanagh et al., 2015). Similar enzyme profiles to those noted for all three strains of this study have been reported by other authors (Medina et al., 2001; Câmara et al., 2019).

**Table 1:** Carbohydrate fermentation profile of *Lactococcus lactis* subsp. *lactis* LA1, *Lactococcus cremoris* subsp. *cremoris* LA10, and *Lactiplantibacillus plantarum* LA30.

Strain	Carbohydrate^a^																			
	GLU	FRU	MAN	LAC	NAG	ESC	GAL	RIB	AMY	ARB	SAL	CEL	MAL	MEL	MNN	SUC	TRE	MLZ	GEN	GNT
LA1	+	+	+	+	+	+	+	+	−	+	+	+	−	−	−	−	+	−	+	−
LA10	+	+	+	+	+	+	−	−	−	−	−	−	−	−	−	−	−	−	−	−
LA30	+	+	+	+	+	+	+	−	+	+	+	+	+	+	+	+	−	+	+	+

Carbohydrates from the API-50 CHL utilized by none of the strains: D-adonitol, D-arabinose, L-arabinose, D-arabitol, L-arabitol, dulcitol, erythritol, glycerol, glycogen, D-fucose, L-fucose, inositol, inuline, 2-ketogluconate, 5-ketogluconate, D-lyxose, methyl-αD-glucopyranoside, methyl-αD-mannopyranoside, methyl-βD-xylopyranoside, D-raffinose, L-rhamnose, D-sorbitol, L-sorbose, starch, D-tagatose, D-turanose, xylitol, D-xylose, and L-xylose.

a Carbohydrate: GLU, D-glucose; FRU, D-fructose; MAN, D-mannose; LAC, lactose; NAG, N-acetylglucosamine; ESC, esculine; GAL, D-galactose; RIB, D-ribose; AMY, amygdaline; ARB, arbutine; SAL, salicine; CEL, D-cellobiose; MAL, D-maltose; MEL, mellibiose; MNN, D-mannitol; SUC, D-sucrose; TRE, D-threhalose; MLZ, D-melezitose; GEN, gentiobiose; GNT, gluconate.

**Table 2:** Enzyme activities measured with the API-ZYM system of *L. lactis* subsp. *lactis* LA1, *L. cremoris* subsp. *cremoris* LA10, and *L. plantarum* LA30.

Strain	Enzyme activity^a^ (nmol)									
	Esterase	Esterase lipase	Lipase	Leu-aryl	Val-aryl	Acid-phos	N-nph	β-gal	β-glu	N-acetyl-β-glu
LA1	5	5	5	0	0	≥40	≥40	5	0	0
LA10	5	5	0	5	0	≥40	≥40	5	0	0
LA30	0	0	0	≥40	≥40	5	20	≥40	20	20

Trypsin, α-chymotrypsin, alkaline phosphatase, cystine arylamidase, α-galactosidase, α-glucuronidase, α-glucosidase, α-mannosidase, and α-fucosidase activities were not detected in any of the strains.

a Activity: Esterase, esterase C4; Esterase lipase, esterase C8; Lipase, lipase C14; Leu-aryl, leucine arylamidase; Val-aryl, valine arylamidase; Acid-phos, acid phosphatase; N-nph, naftol-AS-BI-phosphohydrolase; β-gal, β-galactosidase; β-glu, β-glucosidase; N-acetyl-β-glu, N-acetyl-β-glucosaminidase.

### Safety assessment of the strains

Table 3 shows the results of the resistance-susceptibility analysis for the 16 antibiotics tested. The MICs recorded ranged from <0.03 μg ml^−1^ to >128 μg ml^−1^. *Lactiplantibacillus plantarum* LA30 showed moderate resistance to tetracycline (32 μg ml^−1^), which matches EFSA′s cut-off value for this antibiotic, and strong resistance to vancomycin (>128 μg ml^−1^), while *L. lactis* LA1 proved to be strongly resistant to rifampicin (>64 μg ml^−1^). Vancomycin resistance in lactobacilli is considered intrinsic (Campedelli et al., 2018), and rifampicin resistance in lactococci as usually being caused by either non-specific mechanisms or mutations in the *rpoB* gene (van Pijkeren and Britton, 2012; Goldstein, 2014).

**Table 3:** Minimum inhibitory concentration of 16 antibiotics to *L. lactis* subsp. *lactis* LA1, *L. cremoris* subsp. *cremoris* LA10, and *L. plantarum* LA30.

Strain	Minimum inhibitory concentration (MIC)															
	Gm	Km	Sm	Nm	Tc	Em	Cl	Cm	Am	Pc	Va	Q-da	Lz	Tm	Ci	Rif
LA1	<0.5^a^	8	16	1	1	0.12	0.06	4	0.25	0.25	0.5	2	2	>64	2	>64
LA10	<0.5	<2	4	<0.5	0.5	0.03	<0.06	2	<0.03	<0.03	<0.25	2	0.5	>64	1	8
LA30	<0.5	8	2	<0.5	32	0.12	2	8	1	4	>128	2	4	0.25	16	4
Cut-offs for *lactococci*^b^	**32**	**64**	**32**	**–**	**4**	**1**	**1**	**8**	**2**	**–**	**4**	**–**	**–**	**–**	**–**	**–**
Cut-offs for *L. plantarum*^a^	**16**	**64**	**nr**	**–**	**32**	**1**	**4**	**8**	**2**	**2**	**nr**	**–**	**–**	**–**	**–**	**–**

Key of antibiotics: Gm, gentamicin; Km, kanamycin; Sm, streptomycin; Nm, neomycin; Tc, tetracycline; Em, erythromycin; Cl, clindamycin; Cm, chloramphenicol; Am, ampicillin; Pc, penicillin G; Va, vancomycin; Q-da, quinupristin-dalfopristin; Lz, linezolid; Tm, trimethoprim; Ci, ciprofloxacin; Rif, rifampicin.

a MIC values are in μg ml^−1^.

b The cut-offs applied were those of EFSA FEEDAP Panel (EFSA Panel on Additives and Products or Substances used in Animal Feed) (2018); nr, not required; −, cut-off not established.

*Lactococcus lactis* LA1 proved to be a GABA producer (6.29 mM), while the other two strains were considered non-producers (<0.64 mM; Redruello et al., 2021). None of the strains produced biogenic amines from the precursor amino acids tested; acting as a control, *E. faecalis* V583 produced 5.20 mM tyramine from tyrosine. Production of GABA and formation of biogenic amines require amino acid-specific decarboxylases and are well-known, strain-specific characters (Ladero et al., 2015).

### Growth and metabolite production

**Figure 1:**
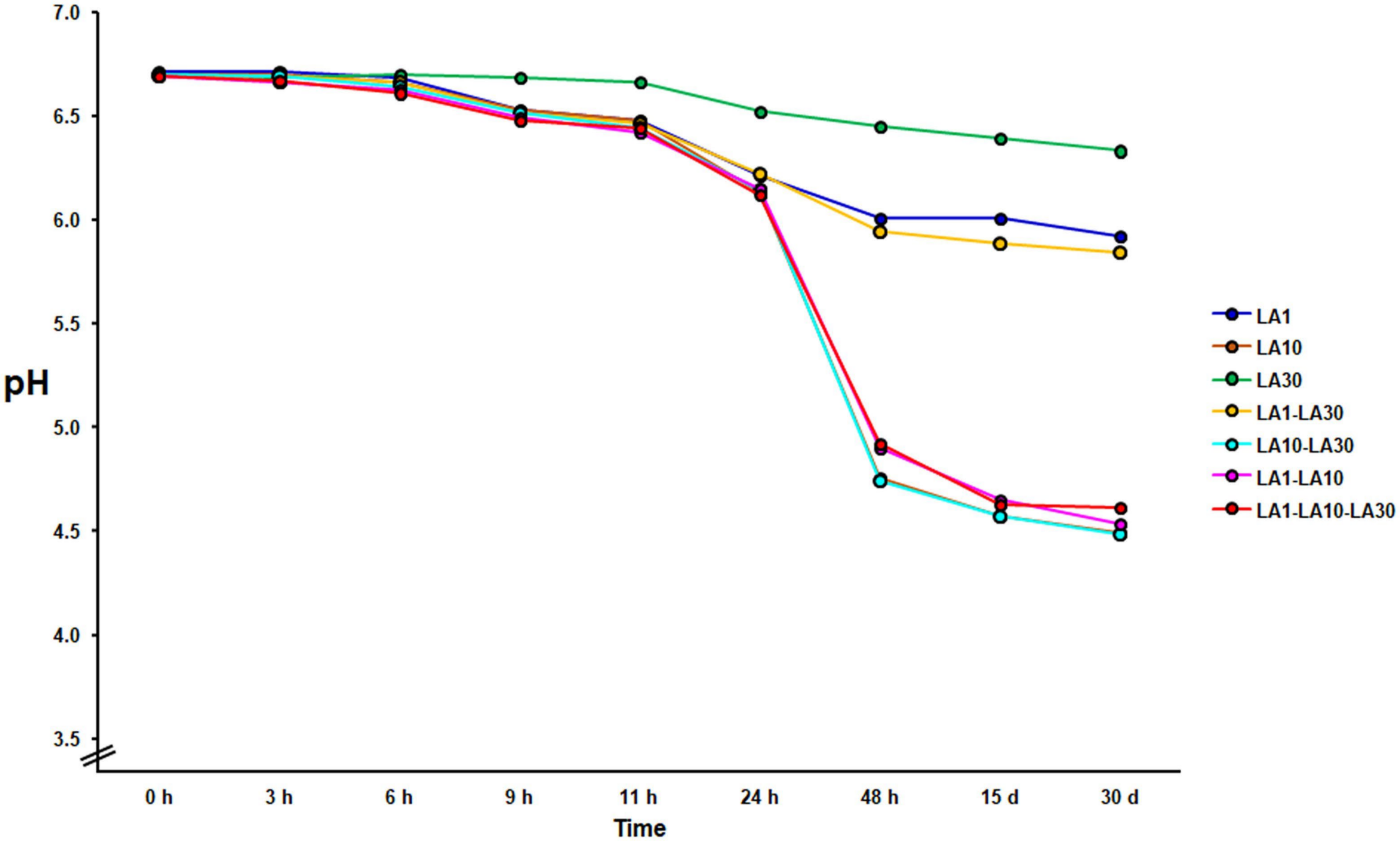
Evolution of the pH during milk fermentation with the individual strains of the consortium and all their mixture combinations. Curves with highly similar slopes were seen in replicate experiments. For the sake of clarity, only one is depicted.

After 48 h incubation at 22°C, only *L. cremoris* LA10, and the combinations containing this strain, coagulated the milk, with the final pH either close to or below 4.6 (4.52 ± 0.33; Figure 1). Under the same incubation conditions, neither *L. lactis* LA1 nor *L. plantarum* LA30, either alone or combined, could coagulate the milk (final pH always >6.0). The fermented milks were stored for up to 1 month at 4°C, during which time a small amount of postacidification was observed (0.21 ± 0.04 pH units).

**Figure 2:**
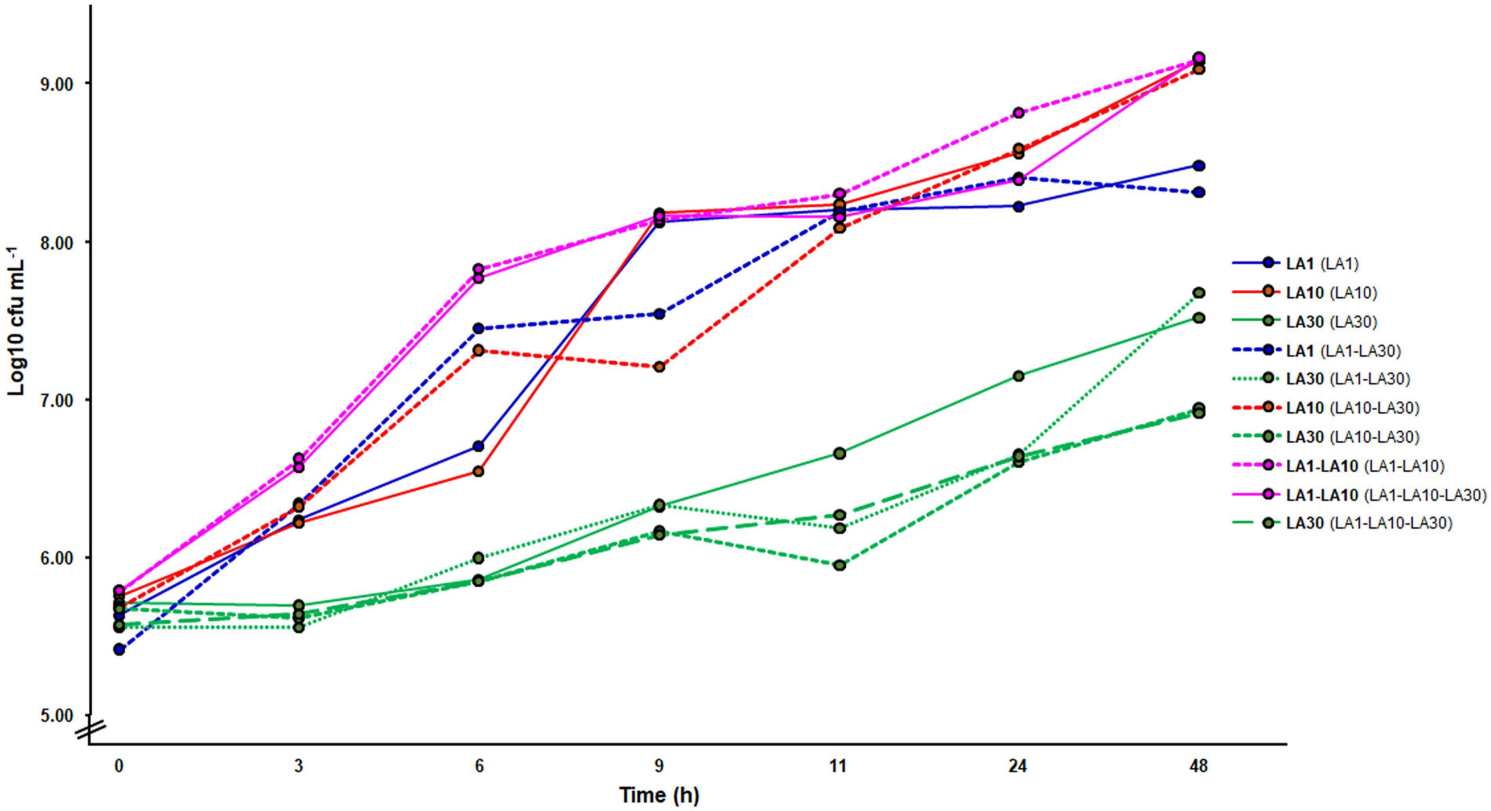
Microbial counts in GM17 (lactococci) and MRS (*Lactiplantibacillus plantarum*) along the fermentation of milk with the individual strains and their mixtures. In bold, the strain(s) counted on each of the curves; in parenthesis, the fermentation from which the counting was made.

Figure 2 summarizes the microbial counts recorded during growth in milk. When grown alone, *L. cremoris* LA10 reached a cell numbers in milk of over 9 log_10_ cfu ml^−1^ (9.14 ± 0.03), while *L. lactis* LA1 and *L. plantarum* LA30 reached cell numbers of 0.5 and 2 log_10_ units lower, respectively (Figure 2). Although not confirmed in this study, as previously determined (Alegría et al., 2010), in the fermented milks made by *L. lactis* LA1 and *L. plantarum* LA30 in combination, equal numbers were assumed for both strains. Alone or combined, counts of *L. plantarum* LA30 were very similar, suggesting the growth of this strain to not be truly stimulated by any companion *Lactococcus*. Similar results were also obtained by real time quantitative PCR, using strain-specific oligonucleotide primers (data not shown).

The individual and combined strains showed various patterns of production/consumption of organic acids and sugars (Table 4). All strains and combinations utilized most of the glucose in milk and part of the lactose. Moreover, *L. cremoris* LA10, and all mixtures including this strain released some galactose to the milk (mean 51.3 ± 16.1 mg 100 ml^−1^). However, this amount was within the range found in cheese and other foods, and low enough to be acceptable even for diets to tackle with classic galactosaemia (Van Calcar et al., 2014). Lactic acid was produced by all three strains, but strongly by *L. cremoris* LA10 and its combinations. Moderate amounts of acetic acid were produced in all milks fermented with *L. lactis* LA1. All –but only– the fermentations involving this latter strain showed citric acid to be readily consumed. Small variations in the fate of other organic acids in the different fermentations were also scored (Table 4).

**Table 4:** Production and consumption of organic acids and sugars during growth in milk at 32°C for 48 h alone or in several combinations of *L. lactis* subsp. *lactis* LA1, *L. cremoris* subsp. *cremoris* LA10, and *L. plantarum* LA30.

Strain–strain mixtures	Organic acid/sugar^a^											
	Orotic	Citric	Pyruvic	Succinic	Lactic	Formic	Acetic	Uric	Hippuric	Lactose^b^	Glucose	Galactose
Uninoculated milk	7.8 ± 2.4	153.6 ± 4.8	0.1 ± 0.1	–	1.2 ± 0.6	–	–	2.4 ± 0.1	2.2 ± 0.1	4874.4 ± 168	10.2 ± 0.2	14.4 ± 0.6
LA1	5.4 ± 1.8	11.4 ± 03.6	4.8 ± 1.8	–	154.8 ± 46.2	–	40.2 ± 11.4	1.8 ± 0.1	1.2 ± 0.6	4695.6 ± 204	0.6 ± 0.2	12.0 ± 4.2
LA10	5.4 ± 0.1	130.2 ± 35.4	0.6 ± 0.1	3.6 ± 0.1	628.8 ± 1.0	0.6 ± 0.1	4.8 ± 0.6	1.8 ± 0.1	–	4173.6 ± 108	0.6 ± 0.1	54.0 ± 0.6
LA30	6.3 ± 1.8	123.6 ± 37.2	0.3 ± 0.1	-	33.6 ± 11.4	–	1.8 ± 0.6	1.8 ± 0.1	1.2 ± 0.5	4663.8 ± 90	0.6 ± 0.1	13.5 ± 4.2
LA1-LA10	6.7 ± 0.1	–	3.0 ± 0.1	1.8 ± 0.1	806.4 ± 12	0.9 ± 0.1	63.6 ± 0.6	2.7 ± 0.1	–	4350.0 ± 108	0.6 ± 0.1	39.0 ± 0.6
LA1-LA30	7.8 ± 0.12	20.4 ± 2.4	7.8 ± 0.4	–	232.2 ± 3.0	–	58.8 ± 3.0	2.4 ± 0.1	2.1 ± 0.2	4792.2 ± 120	0.6 ± 0.1	18.6 ± 0.6
LA10-LA30	6.6 ± 0.1	157.8 ± 1.2	2.4 ± 0.6	4.8 ± 0.6	775.2 ± 1.0	–	7.8 ± 0.6	2.4 ± 0.1	–	4261.2 ± 540	0.6 ± 0.1	73.0 ± 0.6
LA1-LA10-LA30	6.9 ± 0.4	–	4.2 ± 0.2	3.0 ± 0.1	823.8 ± 3.0	0.6 ± 0.1	63.0 ± 3.0	2.7 ± 0.1	–	4276.8 ± 246	0.6 ± 0.1	39.6 ± 1.8

–, not detected.

a Average results of three independent assays are reported in mg 100 ml^−1^.

b Experimental results are being reported (the system is overloaded with the actual content).

Among the 12 volatile compounds detected in the fermented milks, only six were quantified by HS/GC/MS (Table 5). In agreement with the utilization of citrate, diacetyl and acetoin were detected mostly in milks fermented by *L. lactis* LA1. Confirming the previous HPLC analysis, the presence of acetic acid was also associated with fermentations involving this strain. Surprisingly, and compared to any other fermented milk, the sample inoculated with all three strains showed 6 times the amount of acetaldehyde, suggesting it may be the result of an interaction between the consortium strains. In bacteria, acetaldehyde can be derived from the metabolism of amino acids, nucleotides or pyruvate (Bongers et al., 2005). However, despite the several biochemical pathways thus available, acetaldehyde is hardly ever detected as a fermentation end-product of LAB species other than those in yoghurt cultures composed of *Lactobacillus delbrueckii* subsp. *bulgaricus* and *Streptococcus thermophilus* (Chen et al., 2017). Although the nature of the interaction between the three components of the consortium leading to increased acetaldehyde production deserves further investigation, this result reinforces the view that some communities can display properties not shown by their individual members (Minty et al., 2013).

**Table 5:** Relative abundance of the volatile compounds produced and quantified during growth in milk at 32°C for 48 h of *L. lactis* subsp. *lactis* LA1, *L. cremoris* subsp. *cremoris* LA10, and *L. plantarum* LA30, each incubated alone or in combination.

Strain–strain mixtures	Volatile compound^a^^,^^b^					
	Acetaldehyde	2-Propanone	Ethanol	Diacetyl	Acetoin	Acetic acid
Uninoculated milk	–	–	–	–	–	–
LA1	67 ± 19	4 ± 2	19 ± 5	5 ± 1	62 ± 10	23 ± 6
LA10	71 ± 4	27 ± 4	122 ± 6	–	7 ± 5	–
LA30	50 ± 32	16 ± 12	33 ± 5	–	4 ± 2	–
LA1-LA10	152 ± 43	11 ± 2	23 ± 5	30 ± 7	123 ± 37	51 ± 20
LA1-LA30	63 ± 29	14 ± 5	11 ± 2	13 ± 6	66 ± 25	25 ± 19
LA10-LA30	100 ± 19	28 ± 9	115 ± 25	–	14 ± 7	–
LA1-LA10-LA30	602 ± 37	11 ± 3	23 ± 4	31 ± 17	144 ± 34	51 ± 20

–, not detected.

a Carbon disulfide, 2-methyl propanal, 2-propanone, 3-methyl butanal, 2-methyl-1-propanol, and 3-methyl-1-butanol were detected in most fermentations but not quantified.

b Results are average of three independent assays.

The flavor components of fermented milks include volatile and non-volatile compounds; some are already present in the starting milk, but most are produced during fermentation (Settachaimongkon et al., 2014; Beltrán-Barrientos et al., 2019). The major volatile compounds commonly include lactic and acetic acids, acetaldehyde, diacetyl, acetoin, 2,3-butanediol, and 2-butanone (Chen et al., 2017). These compounds are mostly generated through glycolysis or *via* the metabolism of citrate. In the presence of a fermentable carbohydrate (e.g., lactose), citrate is utilized by *L. lactis* subsp. *lactis* biovar *diacetylactis* as a secondary means of generating proton motive force (PMF; Drider et al., 2004). By increasing the intracellular redox potential of the cell, enhancing disulfide bond formation and reducing cofactor reoxidation (Waché et al., 2002), enhanced PMF promotes cell growth.

### Genome analysis

Whole-genome sequencing (WGS) and analysis is currently considered as the gold standard of genetic characterization of microorganisms, including LAB (Liu et al., 2005). The general features of the genome sequences of the three strains of this study are summarized in Table 6. These proved to be similar to those on the literature for strains of the corresponding LAB species. A large number of corrupted genes was found in the genome of all three (59, 105, and 15 for *L. lactis* LA1, *L. cremoris* LA10, and *L. plantarum* LA30, respectively). Conventional PCR amplification, sequencing and sequence comparison around the corrupted positions in seven single-copy genes (three from *L. lactis* LA1 and four from *L. plantarum* LA30) confirmed all mutations identified by genome sequencing. Even though LAB are well known to show gene decay as part of their adaptation (domestication) to the milk environment (Callanan et al., 2008; Cavanagh et al., 2015), one of the most striking features of the sequenced strains was the large number of corrupted genes possessed by all three. In particular, the genome of *L. cremoris* LA10 was so eroded that, as suggested for other *L. cremoris* strains (Wels et al., 2019), it would seem to now be restricted to the dairy environment.

**Table 6:** General features of the genome sequences of *L. lactis* subsp. *lactis* LA1, *L. cremoris* subsp. *cremoris* LA10, and *L. plantarum* LA30 strains from the fermented milk consortium.

Property/encoding genes^a^	*L. lactis* subsp. *lactis* LA1	*L. cremoris* subsp. *cremoris* LA10	*L. plantarum* LA30
Genome size (bp)	2,433,628	2,387,995	3,225,998
G + C content	34.99	35.52	44.39
No. of contigs	104	202	253
Contig N50	61,997	27,520	8,2,412
No. of coding sequences	2,558	2,629	3,271
Proteins with functional assignments	1,986	2,007	1,802
Hypothetical CDS	571	621	1,469
No. of PATRIC subsystems	204	204	169
Antibiotic Resistance (PATRIC/CARD)	26/2	27/2	25/0
Acquired resistance to antibiotics	–	–	–
Virulence Factors (VFDB/Victors)	1/7	1/7	0/0
Penicillin binding proteins	2	1	4
Efflux-related proteins	27	25	28
Resistance to heavy metals	7	6	6
Distinct rRNA operons (23S + 16S + 5.8S)	4 (1 + 1 + 2)	4 (1 + 1 + 2)	3 (1 + 1 + 1)
tRNA molecules	51	50	57
Transporter (TCDB)	73	77	14
Proteases	15	17	18
Caseinolitic proteases (PtrP-like)	–	1	–
Peptidases	26	26	33
Transposases/mobile elements	26	23	39
Glycosil hydrolases	35	12	24
Phage-derived proteins	164	123	25
Tentative functional integrated phages	1	1	2
Plasmid replication proteins	6	11	1
CRISPR loci	–	–	-
Bacteriocins	Lactococcin A	–	Plantaricin F
Toxin-antitoxin systems	Exfoliative toxin A	Exfoliative toxin A	YdcE, HigB-HigA, YefM

a Only complete, non-corrupted genes were included.

#### Identification by genome data

The taxonomic identification of the strains by *in silico* dDDH and OrthoANIu analyses of the genomic data against the type strains of *Lactococcus* spp. and *Lactiplantibacillus* spp. confirmed the strains as belonging to *L. lactis* subp. *Lactis* (LA1), *L. cremoris* subsp. *cremoris* (LA10), and *L. plantarum* (LA30), respectively. LA1 showed the highest dDDH and OrthoANIu values to *L. lactis* subsp. *lactis* biovar *diacetylactis* GL2^T^ (Supplementary Table S1); thus, LA1 was considered to belong to the *diacetylactis* biovar; phenotypic data were then supported by genome analysis (see below).

#### Proteolytic system and amino acid catabolism

The efficient growth of LAB in milk requires the presence of an extracellular, cell envelope-associated, caseinolytic proteinase (PrtP, PrtB, PrtH, or equivalent) to meet the strong demand for nitrogen (Liu et al., 2010). A gene encoding a 1960 amino acids long PrtP type II pre-pro-proteinase showing 99% identity to that of *L. cremoris* subsp. *cremoris* SK11 (De Vos et al., 1989) was identified in the genome of *L. cremoris* LA10. As expected, downstream of the proteinase gene, oppositely orientated and 320 bp away, an ORF encoding a peptidylprolyl isomerase-like lipoprotein of 299 amino acids was detected, known as PrtM (Haandrikman et al., 1989). This acts as a chaperone that converts the pro-proteinase into the active protease. The ORF and its encoded protein were identical to those reported in other strains of this species (Haandrikman et al., 1989). The two genes, *prtP* and *prtM*, were found in a 19,727-bp-long contig associated with sequences involved in plasmid replication. With minor rearrangements, the whole contig sequence showed extensive homology to lactococcal proteinase plasmids, particularly to segments of the long plasmids pDRC3A (103.88 kbp) from *L. lactis* subsp. *lactis* DRC3 (NZ_CP064836.1) and pJM3A (75.81 kbp) from *L. cremoris* subsp. *cremoris* JM3 (NZ_CP016737.1). Although a plasmid location of the proteinase genes in *L. cremoris* LA10 is strongly suggested, the proteinase activity proved to be stable and proteinase-negative variants never observed.

No genes encoding an equivalent proteinase and its maturation protein were identified in the genome of *L. lactis* LA1 or *L. plantarum* LA30. Consequently, these strains grew more slowly in milk that did *L. cremoris* LA10. *Via* the action of the proteinase, *L. cremoris* LA10 could provide casein nitrogen to its partners in mixed fermentations; certainly it has long been recognized that the proteolytic activity of proteinase-positive strains enables non-proteolytic variants to reach high cell densities in milk (Juillard et al., 1996). However, great differences in proteolytic activity have been reported among proteinase-positive strains (Kieliszek et al., 2021), and the stimulation of the growth of proteinase-negative strains by their proteinase-positive counterparts has recently been shown to be strain-specific (Canon et al., 2021). Indeed, the growth of *L. plantarum* LA30 in the present work did not seem to be stimulated by *L. cremoris* LA10. Despite this, *L. plantarum* might be stimulated by other amino acid-derived compounds, such as the GABA produced from glutamate by *L. lactis* LA1, as has been reported for *Leuconostoc mesenteroides* in a traditional cheese starter system (Erkus et al., 2013).

Complex repertoires of genes involved in protein and peptide degradation and subsequent amino acid catabolism and flavor formation were detected in all three strains. Indeed, all strains possessed complex repertoires of genes coding for proteases (14–17 per strain), peptidases (including amino-, carboxy-, and endo-peptidases; 26–31), different amino acid/di−/tri−/oligo-peptide transporters (15–29), aminotrasferases and transaminases (14–21). This genetic repertoire suggests that these strains might be used in starter cultures for other dairy products. Most amino acid-derived volatile compounds, however, may only have a significant impact on the sensory profile of long-ripened fermented dairy products, such as cheese (Smid and Kleerebezem, 2014).

#### Lactose utilization

To grow efficiently in milk, LAB also require a proficient lactose utilization system (this sugar is the main carbon source in this medium). The genetics of lactose utilization by LAB species and strains is complex but rather well known (for a recent review see Iskandar et al., 2019). Lactose is metabolized by LAB either *via* the Leloir or tagatose-6-phosphate pathways. Lactose catabolism in LAB normally proceeds by the Leloir pathway, whereas the tagatose-6-phosphate pathway is mostly restricted to strains of *Lactococcus* spp., *Lacticaseibacillus casei* and *Lacticaseibacillus paracasei* species (Iskandar et al., 2019). However, gene content variation in terms of the components of these lactose pathways in LAB strains of different origin has been repeatedly reported (Passerini et al., 2013). Gene clusters encoding proteins involved in the two pathways were identified in the genome of *L. lactis* LA1 and *L. cremoris* LA10, while in *L. plantarum* LA30 only genes of the Leloir pathway were found (Supplementary Table S2).

**Figure 3:**
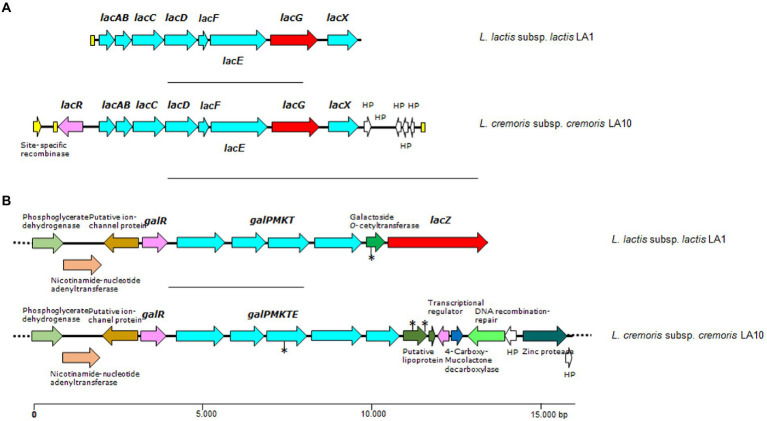
Genetic organization of lactose/galactose gene clusters (**A** and **B**) found in the genome of *L. lactis* LA1 and *L. cremoris* LA10 isolated from a naturally fermented milk. Color code: in red, genes coding for β-galactosidases, *lacZ*, or phospho-β-galactosidase, *lacG*; in pale blue, genes and components of carbohydrate transporter and metabolism; in yellow, open reading frames (ORFs) encoding transposases or mobilization proteins; in purple, genes coding for regulatory proteins. Yellow boxes indicate DNA repeats, and the asterisks denote ORFs containing mutations disrupting the gene. Dotted lines indicate that the contig extends beyond the depicted area.

In the Leloir pathway, lactose is internalized by the product of *galP*, and then hydrolysed by a β-galactosidase (encoded by *lacZ*; Iskandar et al., 2019). The genome of *L. plantarum* LA30 showed three genes encoding lactose/galactose permeases, of which one was associated with a β-galactosidase-encoding gene (*lacZ*). Three other β-galactosidase genes were found scattered throughout the *L. plantarum* genome, one of which was heterodimeric (*lacLM*). The genome of *L. lactis* LA1 and *L. cremoris* LA10 showed a single locus harboring canonical genes of the Leloir pathway for lactose and galactose (*galPMKTE*; Figure 3). A β-galactosidase-encoding gene was located close to the Leloir pathway genes in the *L. lactis* LA1 genome. No *lacZ* gene was found in the genome of *L. cremoris* LA10. In this latter strain, the sequence encoding the galactokinase gene (*galK*) contained a mutation splitting the ORF into two halves. This would explain the inability of *L. cremoris* LA10 to utilize intracellular galactose in the API-50 test, and the presence of galactose in the supernatant of all milk samples fermented with this strain. Although a galactose permease and several low-affinity galactose PTS systems have been shown to be active in *L. lactis* and *L. cremoris* (and probably in other LAB species; Solopova et al., 2018), the internalization of galactose from milk by LA1 and LA30 strains in the presence of an extremely high amount of lactose might be repressed.

A single locus containing all required genes for the tagatose-6-phosphate pathway was identified in the genome of both lactococcal strains. Not surprisingly, each was associated with plasmid-encoded traits. Within this locus, an operon-like structure harboring genes coding for galactose-6-phosphate isomerase (*lacAB*), tagatose-6-phosphate kinase (*lacC*), tagatose 1,6-bisphosphate aldolase (*lacD*), the lactose-specific components of the PTS system (*lacFE*), 6-phospho-beta-galactosidase (*lacG*), and LacX (*lacX*), were identified, plus the oppositely transcribed gene encoding the pathway regulator (*lacR*; Figure 3).

In *L. cremoris* LA10, lactose must be utilized *via* the tagatose-6-phosphate pathway since it lacks the *lacZ* gene and has a mutation in *galK*. However, in *L. lactis* LA1 the gene collections required for both pathways appear to be complete, suggesting them to be functional.

#### Citrate metabolism

Diacetyl (2,3-butanedione) and acetoin (3-hydroxy-2-butanone) are creamy and buttery flavor compounds formed from pyruvate, which are pivotal in many dairy products (Afshari et al., 2020).

**Figure 4:**
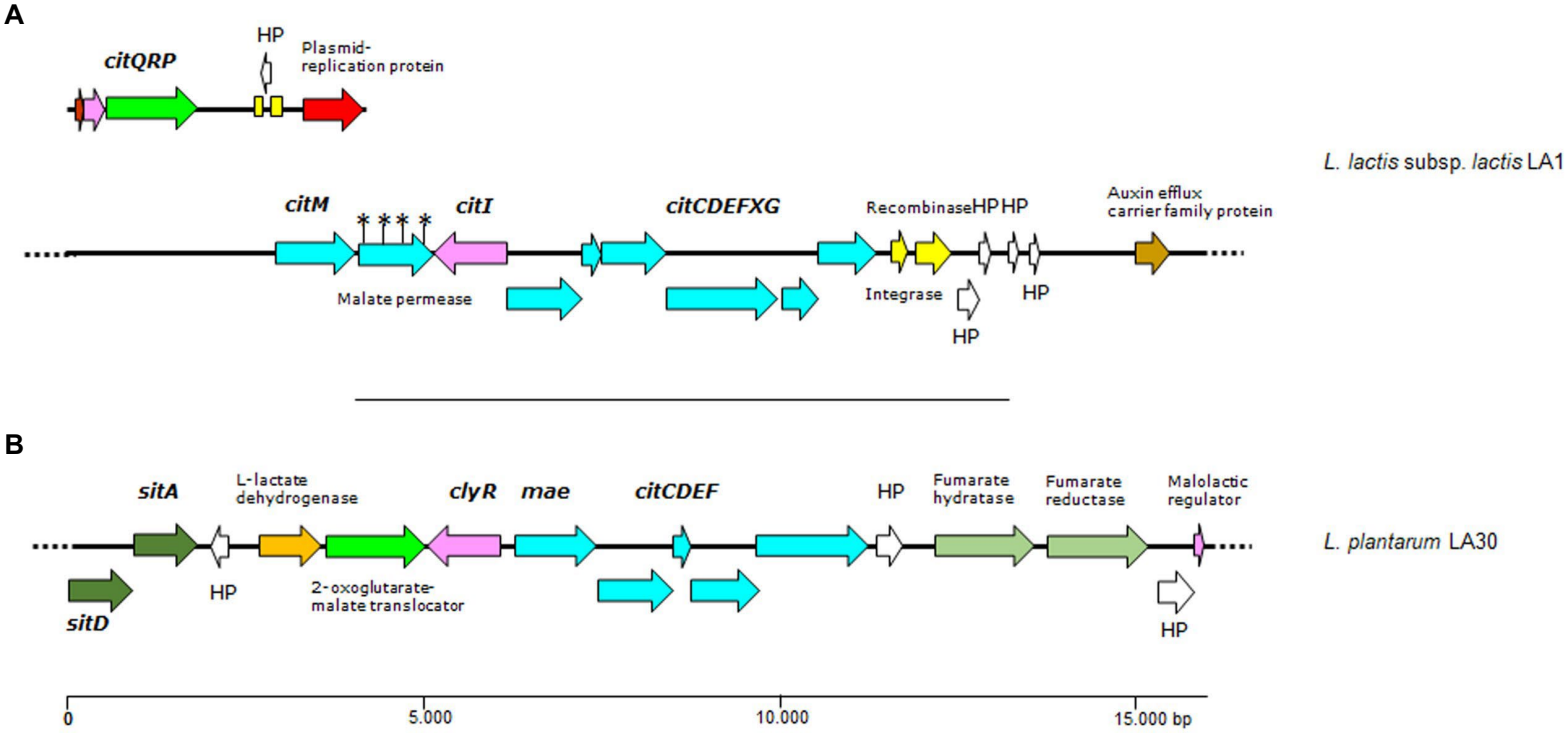
Genetic organization of citrate gene clusters (**A** and **B**) found in contigs of the genome of *L. lactis* LA1 and *L. plantarum* LA30. Color code: in pale blue, genes of the citrate operons (*citM-citCDEFXG*) and (*mae-citCDEF*); in green, genes involved in transport, including a plasmid-borne citrate permease (*citP*); in pink, genes encoding regulatory proteins; in yellow, genes encoding mobilization proteins; in white, genes encoding hypothetical proteins; any other color, other genes. Yellow boxes indicate DNA repeats, and the asterisks denote ORFs dirupted due to mutations disrupting a gene. Dotted lines as in Figure 3.

In agreement with the citrate-utilizing phenotype of *L. lactis* subsp. *lactis* biovar *diacetylactis* LA1, two gene clusters (one plasmid-located and one in the chromosome) involved in citrate fermentation were identified in its genome. The plasmid-borne *citQRP* operon encodes the only citrate transporter system identified to date in lactococci of the *diacetylactis* biovar., CitP (García-Quintáns et al., 2008), as well as the transcriptional regulator CitR, and a putative protein CitQ (López de Felipe et al., 1995). *citP* and its accompanying genes were identified as associated with genes coding for plasmid replication functions in a contig of 7,270 bp, suggesting a plasmid location (Figure 4). The whole sequence was identical to a major part of the citrate plasmids from *L. lactis* subsp. *lactis* biovar *diacetylactis*, i.e., pSD96_04 (CP043528.1), pAH1-6 (CP093419.1) and pCRL1127 (AF409136.1). Compared to these plasmids, the contig in *L. lactis* LA1 lacked the complete nucleotide sequence of a 999 bp-long IS982-like element. This was found in a larger contig, and might have prevented the assembly software allocating a copy to the citrate plasmid. No such an equivalent cluster was identified in either *L. cremoris* LA10 or *L. plantarum* LA30 genomes.

The formation of pyruvate from citrate involves the chromosomally clustered genes *citM-citI-citCDEFXG*, which encode the α-, β-, and γ-citrate lyase subunits (CitF, CitE, and CitD, respectively), the CitC, CitX and CitG auxiliary proteins, and the oxaloacetate decarboxylase CitM (Passerini et al., 2013). Upstream of *citC* is a divergent open reading frame coding CitI, which may regulate the expression of CitP and the assembly of the citrate lyase complex (Martín et al., 2005). All these genes and others encoding key enzymes involved in diacetyl formation (α-acetolactate synthase, lactate dehydrogenase and α-acetolactate decarboxylase) were identified in the genome of *L. lactis* LA1 (Figure 4). These genes support genetically the diacetyl- and acetoin-producing phenotype of this strain. Again, no genes involved in citrate metabolism were identified in *L. cremoris* LA10, and only genes coding for the citrate lyase complex were detected in *L. plantarum* LA30 (Figure 4).

#### Safety assessment

Beyond genes encoding multidrug efflux pumps (Table 6) and proteins involved in heavy metal homeostasis (around seven in each strain), no genes associated with virulence or pathogenicity factors were detected in any of the strains. Two consecutive ORFs, the products of which were identical to the N- and C-terminal parts of a ribosomal protection protein of the TetM/TetW/TetO/TetS family involved in tetracycline resistance, were observed in the genome of *L. plantarum* LA30. These ORFs resulted from an internal mutation of the tetracycline resistance gene, which gave rise to two non-functional protein halves. This gene was located on a 90,000 bp-long contig, suggesting it to be chromosomally encoded. Gene context analysis showed it to have no relationship with sequences involved in mobilization.

Mutations in the *rpoB* gene, which encodes the β subunit of the bacterial RNA polymerase, are known to be related to resistance to rifampicin in *L. lactis* (Goldstein, 2014). Analysis of the *L. lactis* LA1 *rpoB* gene proved to contain mutations leading to two amino acid changes (D489V and H499N). However, the same amino acid replacements were found in the deduced RpoB sequence from the highly susceptible *L. cremoris* LA10 strain. Thus, the cause of this resistance in the LA1 strain remains to be determined. Certain multidrug resistance (MDR) transporters devoted to cell detoxification have also been implicated in rifampicin resistance in lactococci (Filipic et al., 2013).

Apart from a glutamic acid decarboxylase gene present in all three strains, and in agreement with the strains having a negative phenotype for the production of biogenic amines, virtually no genes coding for decarboxylases that might act on amino acids were detected. The exception was a gene in *L. plantarum* LA30 coding for a lysine decarboxylase-family protein. This gene was detected in the vicinity of that coding for L-*O*-lysylphosphatidylglycerol synthase, an enzyme involved in the synthesis of the major bacterial membrane phospholipid (Carey et al., 2022). Lysine carboxylases convert lysine to 1,5-pentanediamine (cadaverine), another well-known cell wall component contributing to maintaining normal bacterial growth (Takatsuka and Kamio, 2004).

## Conclusion

In summary, this work reports the phenotypic and genomic characterization of three strains of different species found as the components of a stable LAB consortium of a NFM. All three strains were susceptible to all important antibiotics, and the inactive TetM/TetW/TetO/TetS-encoding gene does not seem to be associated to any spreading feature, such as plasmids, insertion sequence (IS) elements or transposons, which, according to EFSA′s recommendations (EFSA FEEDAP Panel (EFSA Panel on Additives and Products or Substances used in Animal Feed), 2018) is considered of no concern. Further, genome analysis detected no gene coding for virulence or pathogenicity factors, and none of the strains produced biogenic amines from precursor amino acids. The stability of the consortium must certainly have an underlying biochemical (and thus genetic) basis, though what this might be has yet to be revealed. The *L. cremoris* LA10 strain was the only one of those examined to have a PrtP type II proteinase capable of digesting milk caseins, which may produce the peptides required to feed its nitrogen demands –and perhaps those of its consortium partners. *Lactococcus lactis* LA1 was found to belong to subsp. *lactis* biovar *diacetylactis*, and to utilize milk citrate to produce secondary C-4 metabolite aroma compounds, such as diacetyl and acetoin –the main contributors to the flavor pattern of this NFM. The role of *L. plantarum* LA30 in the consortium, and its interactions with its lactococcal companions, remains to be determined. The consortium as a whole or its individual strains might have a use as a starter or as starter components for dairy fermentations.

## Data availability statement

The data presented in the study are deposited in GenBank database under under Bioproject PRJNA876833 and BioSample accession numbers SAMN30673470 (L. lactis LA1), SAMN30673504 (L. cremoris LA10), and SAMN30673505 (L. plantarum LA30).

## Author contributions

JR and LV: experimental work, analysis of results, and draft editing. AF: conceptualization of research, analysis of results, and manuscript revision and editing. BM: conceptualization of research, analysis of results, revision and editing of the manuscript, funding acquisition, and material resources. All authors contributed to the article and approved the submitted version.

## Funding

This research was supported by projects from the Spanish Ministry of Science and Innovation (PID2019-110549RB-I00/AEI/10.13039/501100011033) and the Principality of Asturias (AYUD/2021/57336). A Ph.D. grant from the Severo Ochoa Program of the Principality of Asturias was awarded to JR (BP19-098).

## Conflict of interest

The authors declare that the research was conducted in the absence of any commercial or financial relationships that could be construed as a potential conflict of interest.

## Publisher’s note

All claims expressed in this article are solely those of the authors and do not necessarily represent those of their affiliated organizations, or those of the publisher, the editors and the reviewers. Any product that may be evaluated in this article, or claim that may be made by its manufacturer, is not guaranteed or endorsed by the publisher.

## Footnotes

^1^ https://www.bioinformatics.babraham.ac.uk/projects/fastqc/

^2^ https://www.bioinformatics.babraham.ac.uk/projects/trim_galore

^3^ https://www.ebi.ac.uk/~zerbino/velvet/

^4^ https://www.patricbrc.org/

^5^ https://cge.cbs.dtu.dk/services/ResFinder/

^6^ https://card.mcmaster.ca/

^7^ http://www.phidias.us/victors/
