# Corrigendum: Phenotype testing, genome analysis, and metabolic interactions of three lactic acid bacteria strains existing as a consortium in a naturally fermented milk

**DOI:** 10.3389/fmicb.2023.1308448

**Published:** 2023-11-02

**Authors:** Javier Rodríguez, Lucía Vázquez, Ana Belén Flórez, Baltasar Mayo

**Affiliations:** ^1^Departamento de Microbiología y Bioquímica, Instituto de Productos Lácteos de Asturias (IPLA), Consejo Superior de Investigaciones Científicas (CSIC), Villaviciosa, Spain; ^2^Instituto de Investigación Sanitaria del Principado de Asturias (ISPA), Oviedo, Spain

**Keywords:** lactic acid bacteria, *Lactococcus lactis*, *Lactococcus cremoris*, *Lactiplantibacillus plantarum*, starters, naturally fermented milk, consortium, genomics

In the published article, there was an error in Table 4 as published. The dilution factor utilized in the UHPLC analysis of sugars and organic acids (parts of sample per parts of diluent 1:6) was not applied. The correction of this omission impacts the absolute values of all compounds analyzed (which should be multiplied by 6), while maintaining their relative concentration. In addition, figures (averages and standard deviations) have all been rounded up to the first decimal. The corrected Table 4 and its caption and footnotes appear below. As a consequence of the update, the sentence on page 5 “Moreover, *L. cremoris* LA10, and all mixtures including this strain released some galactose to the milk (mean 8.6 ± 2.7 mg 100 ml−1)” should read “Moreover, *L. cremoris* LA10, and all mixtures including this strain released some galactose to the milk (mean 51.3 ± 16.1 mg 100 ml−1)”.

**Table 4:** Production and consumption of organic acids and sugars during growth in milk at 32°C for 48 h alone or in several combinations of *L. lactis* subsp. *lactis* LA1, *L. cremoris* subsp. *cremoris* LA10, and *L. plantarum* LA30.

**Strain-strain mixtures**	**Organic acid/sugar** ^**a**^											
	**Orotic**	**Citric**	**Pyruvic**	**Succinic**	**Lactic**	**Formic**	**Acetic**	**Uric**	**Hippuric**	**Lactose** ^b^	**Glucose**	**Galactose**
Uninoculated milk	7.8 ± 2.4	153.6 ± 4.8	0.1 ± 0.1	–	1.2 ± 0.6	–	–	2.4 ± 0.1	2.2 ± 0.1	4874.4 ± 168	10.2 ± 0.2	14.4 ± 0.6
LA1	5.4 ± 1.8	11.4 ± 03.6	4.8 ± 1.8	–	154.8 ± 46.2	–	40.2 ± 11.4	1.8 ± 0.1	1.2 ± 0.6	4695.6 ± 204	0.6 ± 0.2	12.0 ± 4.2
LA10	5.4 ± 0.1	130.2 ± 35.4	0.6 ± 0.1	3.6 ± 0.1	628.8 ± 1.0	0.6 ± 0.1	4.8 ± 0.6	1.8 ± 0.1	–	4173.6 ± 108	0.6 ± 0.1	54.0 ± 0.6
LA30	6.3 ± 1.8	123.6 ± 37.2	0.3 ± 0.1	–	33.6 ± 11.4	–	1.8 ± 0.6	1.8 ± 0.1	1.2 ± 0.5	4663.8 ± 90	0.6 ± 0.1	13.5 ± 4.2
LA1–LA10	6.7 ± 0.1	–	3.0 ± 0.1	1.8 ± 0.1	806.4 ± 12	0.9 ± 0.1	63.6 ± 0.6	2.7 ± 0.1	–	4350.0 ± 108	0.6 ± 0.1	39.0 ± 0.6
LA1-LA30	7.8 ± 0.12	20.4 ± 2.4	7.8 ± 0.4	–	232.2 ± 3.0	–	58.8 ± 3.0	2.4 ± 0.1	2.1 ± 0.2	4792.2 ± 120	0.6 ± 0.1	18.6 ± 0.6
LA10-LA30	6.6 ± 0.1	157.8 ± 1.2	2.4 ± 0.6	4.8 ± 0.6	775.2 ± 1.0	–	7.8 ± 0.6	2.4 ± 0.1	–	4261.2 ± 540	0.6 ± 0.1	73.0 ± 0.6
LA1-LA10-LA30	6.9 ± 0.4	–	4.2 ± 0.2	3.0 ± 0.1	823.8 ± 3.0	0.6 ± 0.1	63.0 ± 3.0	2.7 ± 0.1	–	4276.8 ± 246	0.6 ± 0.1	39.6 ± 1.8

–, not detected.

^a^Average results of three independent assays are reported in mg 100 ml^−1^.

^b^Experimental results are being reported (the system is overloaded with the actual content).

The authors apologize for this error and state that this does not change the scientific conclusions of the article in any way. The original article has been updated.

