# Supplementary material for: Phenotype testing, genome analysis, and metabolic interactions of three lactic acid bacteria strains existing as a consortium in a naturally fermented milk
Source: Front Microbiol. 2022 Sep 23;13:1000683. doi: 10.3389/fmicb.2022.1000683 (PMC9539746; doi:10.3389/fmicb.2022.1000683)
Supplement: Supplementary file 1 [file Data_Sheet_1.PDF]

Table S1.- Genomic distance analysis of *Lactococcus* species and subspecies type strains with the sequenced *Lactococcus* strains of this study. Results from digital DNA-DNA hybridization (dDDH) are shown below the self-comparison diagonal, and results from average nucleotide identity (OrthoANI) are shown above the diagonal. Usual species thresholds are 70% for dDDH and 95% for ANI.

	***L. lactis* LA1**	***L. cremoris* LA10**	***L. lactis* ssp. *lactis* ATCC 19435^T**	***L. lactis* ssp. *lactis* bv. *diacetylactis* GL2^T**	***L. lactis* ssp. *hordniae* NBRC 100931^T**	***L. cremoris* ssp. *cremoris* NBRC 100676^T**	***L. cremoris* ssp. *tructae* DSM 21502^T**	***L. allomyrinae* KACC 19319^T**	***L. hircilactis* DSM 28960^T**	***L. taiwanensis* NBRC109049^T**
***L. lactis* LA1**		87.47	**98.77**	**<u>99.50</u>**	**97.64**	86.98	86.46	76.30	73.27	78.98
***L. cremoris* LA10**	34.3		87.72	87.41	87.03	**<u>98.86</u>**	**97.81**	75.66	72.70	77.98
***L. lactis* ssp. *lactis* ATCC 19435^T**	**89.1**	34.1		98.59	97.61	87.00	86.64	75.96	73.32	79.02
***L. lactis* ssp. *lactis* bv. *diacetylactis* GL2^T**	**<u>95.3</u>**	33.0	87.9		97.78	86.45	86.51	75.82	72.99	78.99
***L. lactis* ssp. *hordniae* NBRC 100931^T**	**81.3**	33.0	79.9	81.6		86.63	86.48	75.97	72.36	78.78
***L. cremoris* ssp. *cremoris* NBRC 100676^T**	32.8	**<u>90.0</u>**	32.7	31.6	31.4		98.03	75.97	72.72	77.85
***L. cremoris* ssp. *tructae* DSM 21502^T**	31.8	**80.4**	31.7	31.4	31.6	83.6		75.89	72.68	77.67
***L. allomyrinae* KACC 19319^T**	22.5	22.9	23.3	21.3	22.9	23.3	22.4		72.20	74.95
***L. hircilactis* DSM 28960^T**	24.8	23.3	24.8	24.6	21.4	25.2	23.0	21.4		71.39
***L. taiwanensis* NBRC109049^T**	23.9	22.1	23.7	21.3	23.7	21.8	21.9	22.2	21.3	

dDDH formula d4 (a.k.a. GGDC formula 2): sum of all identities found in HSPs divided by overall HSP length (Meier-Kolthoff and Göker, 2019).

OrthoANI was calculated using the OrthoANIu algorithm, an improved iteration of the original algorithm, which uses USEARCH instead of BLAST (Yoon et al., 2017).

In bold, dDDH and OrthoANI values higher than current accepted thresholds for the species level obtained for strains LA1 and LA10 (>70% and >95%, respectively); underlined, the highest values.

Table S1B.- Genomic Distance Analysis of *Lactiplantibacillus* species type strains with the sequenced *L. plantarum* LA30 of this study. Results from digital DNA-DNA hybridization (dDDH) are shown below the self-comparison diagonal, and results from average nucleotide identity (OrthoANI) are shown above the diagonal.

	**LA30**	***L. plantarum* ATCC 14917^T**	***L. argentoratensis* DSM 16365^T**	***L. pentosus* DSM 20314^T**	***L. paraplantarum* DSM 10667^T**
**LA30**		**99.00**	**95.61**	80.25	86.50
***L. plantarum* ATCC 14917^T**	**92.3**		95.60	80.06	83.23
***L. argentoratensis* DSM 16365^T**	62.5	62.9		79.99	85.98
***L. pentosus* DSM 20314^T**	24.5	23.8	24.7		79.96
***L. paraplantarum* DSM 10667^T**	32.3	31.1	31.1	24.2	

dDDH formula d4 (a.k.a. GGDC formula 2): sum of all identities found in HSPs divided by overall HSP length (Meier-Kolthoff and Göker, 2019).
OrthoANI was calculated using the OrthoANIu algorithm, an improved iteration of the original algorithm, which uses USEARCH instead of BLAST (Yoon et al., 2017).
In bold, dDDH and OrthoANI values higher than current accepted thresholds for the species level obtained for strain LA30 (>70% and >95%, respectively); underlined, the highest values.
