# Supplementary material for: Phenotype testing, genome analysis, and metabolic interactions of three lactic acid bacteria strains existing as a consortium in a naturally fermented milk
Source: Front Microbiol. 2022 Sep 23;13:1000683. doi: 10.3389/fmicb.2022.1000683 (PMC9539746; doi:10.3389/fmicb.2022.1000683)
Supplement: Supplementary file 2 [file Data_Sheet_2.PDF]

Table S2. Presence of catabolic genes in the genome of *L. lactis* subsp. *lactis* LA1, *L. cremoris* subsp. *cremoris* LA10, and *L. plantarum* LA30 involved in growth in milk and formation of flavour (taste and aroma) compounds.

Protein/description	Gene/operon	Function	Strain		
			LA1	LA10	LA30
**Proteases**					
ATP-dependent zinc metalloprotease	*ftsH*	Cleavage of transmembrane sequences, cell division	1	1	1
CAAX amino-terminal protease	-	-	-	1	1
Cell wall-bound caseinolytic proteinase	*prtP*	Digestion of milk caseins	-	1	-
Clp protease	*clpCEPX*	Turnover of cellular proteins	1	1	1
ClpCP protease substrate adapter protein	*mecA*	Activates protein degradation by ClpCP complex	1	1	1
DegP/HtrA serine protease	*htrA*	Clearance of denatured or aggregated proteins from the inner-membrane and periplasmic space	1	1	1
Hsl protease	*hslUV*	ATP-binding and subunits	-	-	1
Intracellular protease	-	-	-	1	1
Late competence processing protease	*comC*	Processing of DNA during uptake	1	1	-
Lon-like protease with PDZ domain	-	Quality-control of proteins	1	1	1
Membrane-associated zinc metalloprotease	*yugP*	Hydrolysis of Met if followed by Ala, Pro, or Ser	-	-	1
Minor extracellular serin protease	-	-	-	1	-
Periplasmic protease	-	-	-	-	1
Phage proteases	-	-	3	2	1
RasP/YluC protease	-	-	1	1	-
Rhomboid serin protease	-	-	1	1	-
SOS-response repressor and protease	*lexA*	Autoproteolysis in SOS response	1	-	1
YmfH protease	*ymfH*		1	1	1
YrrO protease	*yrrO*	-	1	1	-
Zinc protease	-	-	1	1	1
**Peptidases**					
Alanyl-alanine peptidase	-	-	-	-	1
Aminopeptidase C	*pepC*	General cysteine aminopeptidase	1	1	2
Aminopeptidase YpdF	*ypdF*	Hydrolyses Xaa-Pro bonds when Xaa is Ala, Asn or Met	1	1	1
D-alanyl-D-alanine carboxypeptidase	*dacA*	Cross-linking of peptidoglycan chains; PBP	2	2	4
Dipeptidase	-	-	2	2	4
Glutamyl aminopeptidase	*pepA*	Aminopeptidase of Asp- and Glu- peptides	1	1	-
Lysyl aminopeptidase	-	-	1	1	1
Methionine aminopeptidase	*pepM*	Release of Met from proteins and peptides	1	1	1
Multimodular transpeptidase-transglycosylase	-	Peptidoglycan biosynthesis, probably PBPs	3	3	2
Muramoyl tetrapeptide carboxypeptidase	-	-	1	1	1
Neutral endopeptidase	-	-	3	2	1
Oligoendopeptidase F	*pepF*	Hydrolysis of 7-17 amino acids long peptides	1	2	2
Peptidase U32 family	-	-	1	1	-
Proline iminopeptidase	*pepI*	Release proline from peptides	-	-	3
Pyrrolidone-carboxyl peptidase	*pcp*	Removal pyroglutamate from proteins and peptides	1	1	-
Signal peptidase I	-	Release of signal peptides	1	1	2

Signal peptidase II	*lspA*	Release of signal peptides from lipoproteins	1	1	1
SprT-family zinc metallopeptidase	*sprT*	-	1	1	1
Tripeptide aminopeptidase	*pepT*	Amino degradation of tripeptides	1	1	1
Xaa-His dipeptidase	*pepV*	Hydrolysis of Xaa-His dipeptides	1	1	1
Xaa-Pro dipeptidase	*pepX*	Release of dipetides when Pro is at the second position	2	2	2
**Lactose, citrate, amino acid, and peptide transporters**					
Amino acid permease, GabP family	*gabP*	Transport of amino acids	-	3	5
Amino acid permease, YdaO	*ydaO*	Transport of amino acids	-	-	1
Arginine/ornithine antiporter ArcD	*arcD*	Transport arginine	2	2	-
Basic amino acid/glutamine ABC transporter	-	Transport of basic amino acids	2	2	4
Cationic ABC amino acid transporter	*yckB*	Transport of basic amino acids	1	1	-
CitP	*citP*	Citrate transporter	1	-	-
Cysteine ABC transporter	-	Transport of cysteine	2	2	-
Di-tripeptide/cation symporter DtpT	*dtpT*	Transport of di- and tripeptides	1	-	1
D-serine/D-alanine/glycine transporter	-	Transporter of serine, alanine, glycine	-	-	4
Glutamate/gamma-aminobutyrate antiporter	*gadC*	Transport glutamate/export GABA	1	1	-
Glutamine ABC transporter	*glnHPQ*	Transport of basic amino acids	1	1	1
Lactose permease	*lacP*	Transport of lactose	1	1	3
Lactose-specific PTS system	*lacABC*	Transport and phosphorylation of lactose	1	1	-
Methionine ABC transporter	-	Transport of Met	1	1	3
Methionine/phosphonates ABC transporter	-	Transport of Met	1	1	-
Na(+)-dependent branched-chain amino acid transporter	*brnQ*	Transport of branched chain amino acids	1	1	5
Nickel/peptides ABC transporter	-	ATP-binding protein	1	2	-
Oligopeptide ABC transporter	*oppABCDF*	Transport of oligopeptides	1	1	1
Proton/glutamate symporter	-	Transport of glutamate	-	-	1
Serine transporter	-	Transport of serine	-	-	1
Tyrosine transporter, NhaC family	*nhaC*	Transport of tyrosine	-	-	2
**Aminotransferases and transaminases**					
Alanine transaminase	-	Arg synthesis, Ala, Asp, Glu metabolism	1	1	-
Aminotransferase	-	Transfer of a α-amino groups between Asp and Glu	-	-	1
Aromatic amino acid aminotransferase gamma	*araT*	Transfer of α-amino groups between aromatic amino acids	1	1	-
Aspartate aminotransferase	*aspAT*	Reversible transfer of a α-amino group between Asp and Glu	2	2	8
Branched-chain amino acid aminotransferase	*bcaT*	Cys, Val, Leu, Iso, Met, synthesis and metabolism, synthesis of secondary metabolites	1	1	1
Glutamine amidotransferase, class 1	*pfpI*	Removal of the ammonia group from glutamine	1	1	2
Glutamine-dependent 2-keto-4-methylthiobutyrate transaminase	-	Cys, Met metabolism, metabolic pathways	-	-	1
Glutamine-fructose-6-P aminotransferase	*glmS*	Ala, Glu, metabolism, metabolic pathways	1	1	1
Histidinol-phosphate aminotransferase	-	Glu, His metabolic pathways	-	1	1
L,D-transpeptidase	-	Cross-linking of peptide stems in peptidoglycans	-	-	2
Multimodular transpeptidase-transglycosidase	-	-	3	3	2
N-acetyl-L,L-diaminopimelate aminotransferase	-	Ala, Asp, Glu, Gly, Ser, Thr metabolism, Lys degradation, pyruvate metabolism	1	1	-
N-acetylornithine aminotransferase	*rocD*	Arg biosynthesis, metabolic pathways, synthesis of secondary metabolites	1	1	1

Phosphoserine aminotransferase	-	Gly, Ser, Thr, Cys, Met metabolism, synthesis of secondary metabolites	1	1	1
Serine-pyruvate aminotransferase	-	Gly, Ser, Thr metabolism	1	-	-
**Dehydrogenases**					
2,3-butanediol dehydrogenase	-	-	2	2	-
Aldehyde dehydrogenase	*adhE*	Fatty acids degradation, amino acids synthesis and degradation, synthesis of secondary metabolites	1	1	2
Alanine dehydrogenase	*ala-dh*	Ala, Asp, Glu metabolism, metabolic pathways	1	-	-
Alcohol dehydrogenase class III	*adh*	Glycolysis, fatty acid degradation, Tyr metabolism, pyruvate metabolism	3	3	5
Aspartate-semialdehyde dehydrogenase	-	Gly, Thr, Cys, Met metabolism, Lys synthesis	1	1	1
D-lactate dehydrogenase	*ldhD*	Synthesis of secondary metabolites	1	1	3
Homoserine dehydrogenase	-	Gly, Ser, Thr, Cys, Met metabolism, Lys synthesis, metabolic pathways	1	1	2
L-lactate dehydrogenase	*ldhL*	Glycolysis, Cys, Met metabolism, synthesis of secondary metabolites	3	4	5
L-lactate dehydrogenase, Fe-S oxidoreductase	*ykgEFG*	Glycolysis, Cys, Met metabolism, synthesis of secondary metabolites	1	-	-
NADP-specific glutamate dehydrogenase	*gdhA*	Arg synthesis, Ala, Asp, metabolism, metabolic pathways	-	-	1
**Lyases**					
Argininosuccinate lyase	-	Arg biosynthesis, Ala, Asp, Glu metabolism, Metabolic pathways, biosynthesis of secondary metabolites	1	1	1
Argininosuccinate synthase	-	Arg biosynthesis, Ala, Asp, Glu metabolism, Metabolic pathways, biosynthesis of secondary metabolites	1	1	1
Citrate lyase complex	*citCDEFG*	Formation of pyruvate from citrate	1	-	1
Cystathionine beta-lyase	*cglB*	Production of sulphur compounds	-	-	1
Cystathionine beta-synthase	*cbs*	Transulfuration from homocysteine to cystathionine	-	-	1
Cystathionine gamma-lyase	*cglA*	Gly, Ser, Met, Cys, Thr metabolism, metabolic pathways	1	1	1
Cystathionine gamma-synthase	*cgs*	Formation of cystathionine from cysteine	1	1	1
D-serine ammonia-lyase	-	Gly, Ser, Thr metabolism, D-Amino acid metabolism, metabolic pathways	-	-	1
S-ribosylhomocysteine lyase	-	Cys, Met metabolism, metabolic pathways	1	1	1
**Esterases/lipases**					
Alpha/beta hydrolase	-	Lipid transport and metabolism	-	1	-
Carboxylesterase	*est*	Wide specificity acting on ester bonds	1	-	-
Esterase/lipase	*estA*	Lipid transport and metabolism	1	-	1
Glycerophosphoryl diester phosphodiesterase	*glpQ*	Glycerophospholipid metabolism	3	2	3
Lipase/Acylhydrolase	-	-	2	2	1
Monoglyceride lipase	-	-	1	1	-
Phosphoesterase	-	Release of membrane-linked proteins with glycosylphosphatidylinositol anchors	2	2	2
Tributyrin esterase	-	Hydrolysis of C2-C16 fatty acids	1	1	-

Highlighted in colour the main enzyme groups and differences in gene content between strains.
